# Development and Positioning Accuracy Assessment of Single-Frequency Precise Point Positioning Algorithms by Combining GPS Code-Pseudorange Measurements with Real-Time SSR Corrections

**DOI:** 10.3390/s17061347

**Published:** 2017-06-09

**Authors:** Miso Kim, Kwan-Dong Park

**Affiliations:** 1 PPSoln Inc., 606 Seobusaet-gil #A-2409, Seoul 08504, Korea; mskim@ppsoln.com; 2 WCSL (World Class Smart Lab) of intelligent vehicle Inha research Lab, Department of Geoinformatic Engineering, Inha University, 100 Inha-ro, Incheon 22212, Korea

**Keywords:** GPS, PPP, SSR, pseudorange, navigation

## Abstract

We have developed a suite of real-time precise point positioning programs to process GPS pseudorange observables, and validated their performance through static and kinematic positioning tests. To correct inaccurate broadcast orbits and clocks, and account for signal delays occurring from the ionosphere and troposphere, we applied State Space Representation (SSR) error corrections provided by the Seoul Broadcasting System (SBS) in South Korea. Site displacements due to solid earth tide loading are also considered for the purpose of improving the positioning accuracy, particularly in the height direction. When the developed algorithm was tested under static positioning, Kalman-filtered solutions produced a root-mean-square error (RMSE) of 0.32 and 0.40 m in the horizontal and vertical directions, respectively. For the moving platform, the RMSE was found to be 0.53 and 0.69 m in the horizontal and vertical directions.

## 1. Introduction

In the past decade or so, Precise Point Positioning (PPP) has received a significant amount of attention in the GNSS community as a new and effective option for precise real-time positioning [1,2]. PPP is a technique in which the user only estimates receiver-specific parameters such as receiver clock offsets and zenith tropospheric delays, whereas the satellite orbits and clocks are provided by a global GPS analysis center [3]. Dual-frequency receivers are usually involved in PPP to remove the ionospheric delay effect. However, in the PPP-Real Time Kinematic (RTK) mode of PPP, one can get tropospheric and ionospheric delays generated from a GNSS network. Thus, PPP-RTK can be defined as a positioning technique in which the user obtains satellite-orbit, -clock, and -bias corrections from a service provider. The ionospheric delay is removed either through ionosphere-free combinations or by applying the total electron content (TEC) information generated from a network. Finally, the tropospheric delay is considered through a stochastic estimation or by utilizing zenith hydrostatic delay (ZHD) and zenith wet delay (ZWD) information available from the network.

One of the most prominent PPP services is the IGS-RTS, which is managed by the International GNSS Service (IGS). RTS stands for real-time service, and thus, real-time streams of satellite orbits and clock-offset corrections can be obtained through the IGS-RTS service. When such corrections are applied to broadcast navigation messages, the accuracies of three-dimensional orbits improve to the level of ~5 cm, and clock offsets are at a level of ~8 cm compared with the European Space Agency/European Space Operations Centre final product [4]. Notable validation studies on IGS-RTS corrections include those by [5,6]. Won [5] developed PPP algorithms and applied IGS-RTS corrections to them. For static positioning, they obtained a level of accuracy of ~10 cm in the horizontal direction compared to RTK when integer ambiguities are estimated as float numbers. For a mobile platform, the absolute accuracy was within the range of 20 to 30 cm. The real-time performance of IGS-RTS was evaluated by comparing the positioning accuracies obtained using IGS-RTS and IGS rapid products [6]. They obtained a positioning accuracy of 20 cm with IGS-RTS. When IGS rapid products were used instead of IGS-RTS, the horizontal accuracy achieved was approximately 40 cm. Thus, with IGS-RTS, the accuracy improved by approximately 50%.

As noted above, several studies have applied IGS-RTS corrections in processing the carrier phase data. However, case studies implementing IGS-RTS corrections to pseudorange measurements are difficult to find. The only publication combined pseudorange observations with highly accurate satellite orbits and clock offsets was [7], which applied IGS precise SP3 and CLK products instead of real-time corrections. In place of ionospheric and tropospheric corrections, however, they used global ionospheric maps for the ionospheric delay and pre-processed stochastic tropospheric delay estimates. For four days’ worth of data, the horizontal and vertical accuracies were in the range of 0.8–1.6 m and 1.6–2.2 m, respectively. When the same data were processed using Differential GPS (DGPS) positioning mode, PPP outperformed DGPS by 20–70%.

In this paper, we developed PPP algorithms that consider real-time State Space Representation (SSR) corrections for GPS pseudorange processing. SSR corrections have been produced by Seoul Broadcasting System, who operates 20-site GNSS network and a GNSMART server. We start with a pseudorange measurement equation and describe how major error sources were modeled with SSR corrections. The SSR data formats, the categories of information contained in SSR messages, and how they are applied in real-time positioning are explained in detail. Finally, static and kinematic positioning results using GPS pseudorange measurements are described.

## 2. Code-Pseudorange Measurement Equation

Pseudorange measurements are the range or distance from the user antenna to each satellite, and are calculated based on the signal transmission time from the satellites to the user. Such observables, however, contain clock offsets at both the satellite and the user receiver, and are thus not exactly “true” or “geometric” ranges. To make things worse, ionospheric and tropospheric delays, in addition to hardware effects and multipath, come into play. To achieve reliable positioning results, a variety of error sources have to be corrected or compensated for using measurement and force models. A general pseudorange measurement equation can be represented by the following equation:

(1) $$p=\rho +c\left(\delta t_{r}-\delta t^{s}\right)+I+T+M+b^{s}+b_{r}+\varepsilon$$

where *p* is the pseudorange measurement, ρ is the three-dimensional distance from the receiver to a satellite, *c* is the speed of light, *δt_r_* is the receiver clock offset, *δt^s^* is the satellite clock offset, *I* is the ionospheric delay, *T* is the tropospheric delay, *M* is a multipath error, *b^s^* is a satellite bias error, *b_r_* is a receiver bias error, and ε is a random error [8].

In the above equation, considerations regarding the multipath error were not included in our positioning algorithm. This is not because its effects are small, but rather because we were unable to find a reliable and real-time multipath correction model to apply to pseudorange measurements. The satellite ephemeris, e.g., the three-dimensional coordinates $\left(x^{s},y^{s},z^{s}\right)$ in WGS-84, and an unknown receiver position $\left(x_{r},y_{r},z_{r}\right)$ are included as $\rho =\sqrt{{\left(x^{s}-x_{r}\right)}^{2}+{\left(y^{s}-y_{r}\right)}^{2}+{\left(z^{s}-z_{r}\right)}^{2}}$ in Equation (1). Both the satellite ephemeris and the satellite clock offsets are broadcast from the satellite and can be extracted from real-time navigation messages. However, the accuracy of broadcast orbit is at the level of 1 to 2 m [9], it is insufficient for use in precise point positioning. Thus, the first priority is for us to correct them with SSR messages, the procedure of which is explained in Section 3.2.1 and Section 3.2.2

Although the satellite hardware bias *b^s^* is included in the navigation message as *T_gd_*, whose value was set up in the satellite manufacturing stage, but the current and up-to-date value should be estimated on the user side because it is a variable quantity [10]. For this reason, the satellite bias is also included in SSR messages, and satellite-dependent values are given as piecewise constants. The ionospheric and tropospheric errors are the two most prominent medium-dependent error sources, and are usually provided at pre-defined grid points. How these three quantities (*b^s^*, *I*, and *T*) are handled with SSR messages is explained in Section 3.2.3, Section 3.2.4 and Section 3.2.5. Now, the remaining error source is the receiver bias, *b_r_*. This quantity applies to every satellite, and thus, it can be estimated as one lumped value with the receiver clock offset, *δt_r_* [11].

The gravitational attraction of the moon and sun causes periodic deformations of the solid earth, which is referred to as earth tides. The combined tidal variation can reach up to 5 and 30 cm in the horizontal and vertical directions, respectively [12]. Earth tides are expressed using Laplace equations, and the second degree of spherical harmonics account for 98% of the total variation [13]. It is advised to consider only second and third degree of spherical harmonics in space geodetic technology using GNSS. Thus, we considered only the second and third degrees. Although a solid earth tide is not included in Equation (1), we corrected its effect by following the procedure suggested by IERS convention [14].

In summary, if the multipath error is neglected in Equation (1), the pseudorange-based measurement equation has four unknowns: three-dimensional receiver coordinates implicit within range ρ, the lump sum of the receiver clock offset *δt_r_* and the receiver hardware bias *b_r_*. In estimating four states, we used an extended Kalman filter (EKF) in addition to the usual weighted least squares (LS). In the case of the EKF, a priori states were obtained through an LS estimation of the first-epoch measurements.

## 3. Real-Time SSR Messages

### 3.1. Overview of SBS-SSR Messages

**Figure 1:**
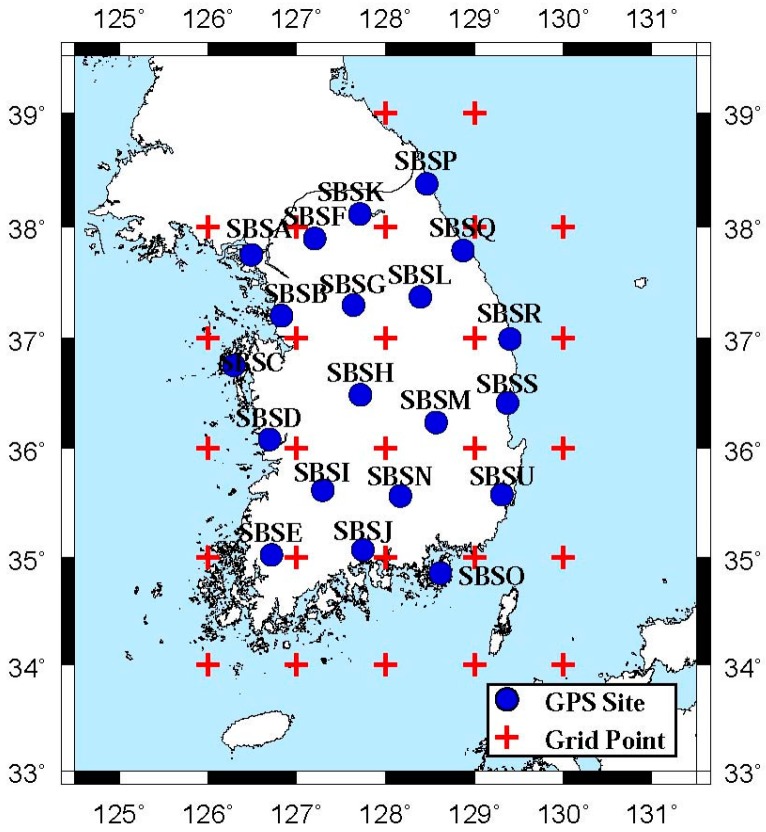
Location of 20-site PPP-RTK network in South Korea. The red crosses denote positions where gridded ionospheric and tropospheric delay corrections are generated in SSR format. The average inter-site distance is ~70 km.

The Seoul Broadcasting System (SBS) of South Korea constructed the PPP-RTK infrastructure and has been testing its performance over the past several months. The infrastructure consists of 20 reference sites, a GNSMART server, and digital multimedia broadcasting (DMB) transmission facilities. Base stations are well distributed throughout the South Korean territory, except Jeju Island (Figure 1), and feed real-time GNSS measurements to the server. The server runs GNSMART software developed by Geo++ of Germany and generates SSR-type correction messages [1]. In addition to broadcasting SSR messages through terrestrial DMB, SBS allows Internet connections to the server.

SBS-SSR messages include corrections regarding five different types of GNSS errors: satellite orbit, satellite clock offsets, satellite biases, ionospheric delays, and tropospheric delays. Message protocols follow the Radio Technical Commission for Maritime (RTCM) services standards for SSR. In the following sub-sections, typical SSR message structures and application procedures are described.

### 3.2. Five Types of SBS-SSR Messages

#### 3.2.1. Satellite Orbit Corrections

In the GPS Standard Positioning Service, real-time broadcast ephemeris is used, and its accuracy is currently at a level of 1 to 2 m [9]. To remove or handle inaccuracies of the broadcast orbits, SBS-SSR provides corrections in the radial, along-track, and cross-track directions. Sample messages are listed in Table A1. The procedures for utilizing SSR orbit messages in correcting broadcast orbits are shown in Equation (2), where ***δX*** indicates corrections to the broadcast orbit ***X_b_*** [4]. The reference system for corrected coordinates ***X_c_*** is the earth-centered earth-fixed coordinate system, WGS-84. The value of ***δX*** should be calculated by applying the inner product of the rotation matrix $\left[e_{radial},e_{along,}\;e_{cross}\right]$ with SSR corrections, ***δO***. An explanation regarding the rotation matrix is given in the next paragraph:

(2) $$X_{c}=X_{b}-\delta X,\;\delta X=\left[e_{radial},\;e_{along},e_{cross}\right]\delta O^{T}$$

SSR orbit corrections, ***δO***, are provided in the radial, along-track, and cross-track directions, and are denoted as $\delta O=\left[\delta O_{radial},\delta O_{along},\delta O_{cross}\right]$ in Equation (2). Direction cosines $\left[e_{radial},e_{along,}\;e_{cross}\right]$ are obtained from Equation (3):

(3) $$e_{along}=\frac{v_{b}^{s}}{\left|v_{b}^{s}\right|},\;e_{cross}=\frac{r_{b}^{s}\times v_{b}^{s}}{\left|r_{b}^{s}\times v_{b}^{s}\right|}\;,\;e_{radial}=e_{along}\times e_{cross}$$

where $r_{b}^{s}$ and $v_{b}^{s}$ are the satellite position and velocity vectors, respectively, calculated using the broadcast ephemeris [15].

#### 3.2.2. Satellite Clock Corrections

Atomic clocks onboard GNSS satellites provide highly stable frequency standards, but the broadcast clock offsets contain errors whose magnitudes are at the level of 5 ns, which corresponds to the range error of 1.5 m. Satellite clock errors are typically modeled according to the second-degree polynomial $ax^{2}+bx+c$, and three coefficients a, b and c are contained in satellite navigation messages, allowing the GNSS user to consider the clock offsets instantaneously. For the purpose of reducing or removing these errors, SBS-SSR messages provide three coefficients, *C*_0_, *C*_1_, and *C*_2_, samples of which are shown in Table A2. In this case, the user can utilize the following formula [15]:

(4) $$\delta t^{s}=\delta t_{b}^{s}-\frac{\delta C}{C}$$

where $\delta t^{s}$ is corrected satellite clock offsets while $\delta t_{b}^{s}$ are non-corrected offsets computed from real-time navigation messages. In Equation (4), *δC* is satellite clock corrections derived from SSR messages, and is computed through the following equation: $\delta C=C_{0}+C_{1}\left(t-t_{o}\right)+C_{2}{\left(t-t_{o}\right)}^{2}$. $C_{0}$, $C_{1}$, and $C_{2}$ are three coefficients for the second-degree polynomial while t and $t_{o}$ correspond to the current epoch and the reference epoch, respectively.

#### 3.2.3. Satellite Bias Corrections

Satellite bias is an error caused by the satellite transmission circuit hardware design, and produces different amounts of signal delay on the pseudorange and carrier phase measurements. In addition, it is dependent on the signal frequency. If not corrected for, the bias influences the satellite clock offsets, causing positioning errors.

SBS-SSR messages provide four (C1, P1, L1, and L2) bias estimates for each satellite, a sample output of which is listed in Table A3. Because the GNSMART server computes signal biases relative to that of the C1 signal, the C1 bias is always set to zero, as shown in the table. Although the bias of C1 is null, its actual value is lumped into satellite clock offsets, as explained in Section 3.2.2. For this reason, we do not apply the C1 bias values as long as the clock offsets from the SBS-SSR are used simultaneously.

#### 3.2.4. Ionospheric Delay Corrections

The amount of GNSS signal delay from the ionosphere depends on the number of electrons existing along the signal path, the corresponding number of which is referred to as the TEC. The unit of the TEC is TECU, which equals 1 × 10^16^ electrons in a 1-m^2^ column along the line of sight. Using a dual-frequency receiver, the ionospheric delay error can almost be completely removed using ionosphere-free combinations. In the case of single-frequency receivers, Klobuchar model parameters broadcast from a satellite can be used in real-time, although the accuracy is unsatisfactory, only compensating ~50% of the total delay [16]. SBS-SSR contains ionospheric delay corrections in terms of the Slant TEC (STEC) at approximately 30 grid points in and around South Korea. As shown in Figure 1, the grid points are made with a resolution of 1° × 1° and cover 34–39° N and 126–130° E. Sample STEC messages are listed in Table A4.

#### 3.2.5. Tropospheric Delay Corrections

The tropospheric delay of GNSS signals is divided into two parts, which are referred to as ‘dry’ and ‘wet’ components. Although a dry or hydrostatic delay amounts to ~90% of the total tropospheric delay, it can be quite accurately determined using surface pressure measurements [17]. A wet delay has a very high spatial and temporal variability and cannot be reliably determined even with accurate surface meteorological observations. Thus, a wet delay should be estimated stochastically.

In the case of SBS-SSR, ZHD and ZWD are provided at pre-defined grid points, eliminating the need for their estimation. After interpolating such delays for the user location, the following equation is applied to obtain the total tropospheric delay along the Line-of-Sight (LoS) direction toward each satellite:

(5) $$\delta T=\delta T_{h}+\delta T_{w}=m_{h}\left(e\right)ZHD+m_{w}\left(e\right)ZWD$$

where *δT, δT_h_* and *δT_w_* are the slant total delay, slant hydrostatic delay, and slant wet delay, respectively. The hydrostatic and wet mapping functions ($m_{h}$ and $m_{w}$) are used to translate the zenith delay to the LoS direction and are functions of elevation angle *e*. Sample tropospheric delays are listed in Table A5.

## 4. Application of SSR Message in Real-Time Positioning

### 4.1. Static Positioning

#### 4.1.1. Test Data and GPS Equipment

For validation of the developed PPP positioning algorithms, we collected GPS C/A measurements at a permanent GNSS site, SBSA, located in the northwest region of South Korea (Figure 1). This site is being operated by SBS, and has a Javad Sigma-G3T receiver and a Javad choke-ring antenna whose ANTEX code is JAVRINGANT-DM. One-second sampling data recorded for a 24-h period on 19 September (DOY 230) and 26 September (DOY 237) of 2015 were taken for the positioning tests. We divided the collected C/A measurements into 48 hourly datasets, and 44 datasets were finally chosen after removing four incomplete datasets. The accuracy of our data processing was evaluated by comparing the estimated coordinates to the precise coordinates of SBSA, which were derived from our routine data processing based on GIPSY-OASIS developed by NASA Jet Propulsion Laboratory. Detailed description of the procedure can be found in [3].

#### 4.1.2. Convergence Time Analysis

The convergence time is a critical factor in evaluating the performance of PPP processing engines. When carrier phase measurements are used, the position coordinates are stochastically estimated along with the integer ambiguities. Through this approach, the convergence times can be measured explicitly by taking the time required for ambiguity fixing. In our case, we do not estimate the integer ambiguities because only pseudorange measurements are involved. For this reason, we had to devise a new criterion for convergence.

**Figure 2:**
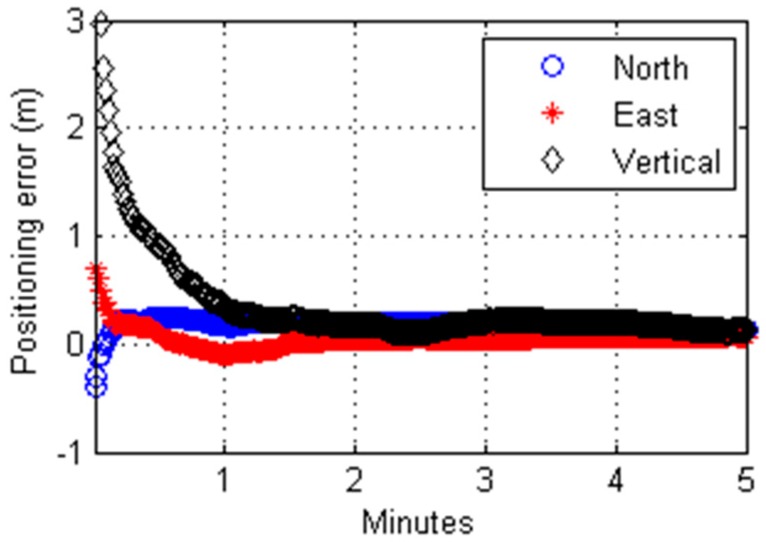
Positioning errors of PPP estimates of the site SBSA on 19 September 2015. These Kalman-filtered estimates are derived from C/A code measurements.

Figure 2 shows a 5-min segment of the estimated coordinates of SBSA for the first hour on DOY 230 of 2015. The positioning accuracy improves quite quickly during the early stage of the estimation process. After about a minute or so, the improvement is either very slow or barely noticeable. This pattern of “fast convergence at the beginning” was found in every dataset we processed. For this reason, we conclude that the rate of change of the estimated position qualifies as a reliable criterion for determining the convergence of a Kalman-filtered solution of a pseudorange-only PPP. The rate of change in the estimated coordinate is referred to as the coordinate rate of change (CROC).

**Figure 3:**
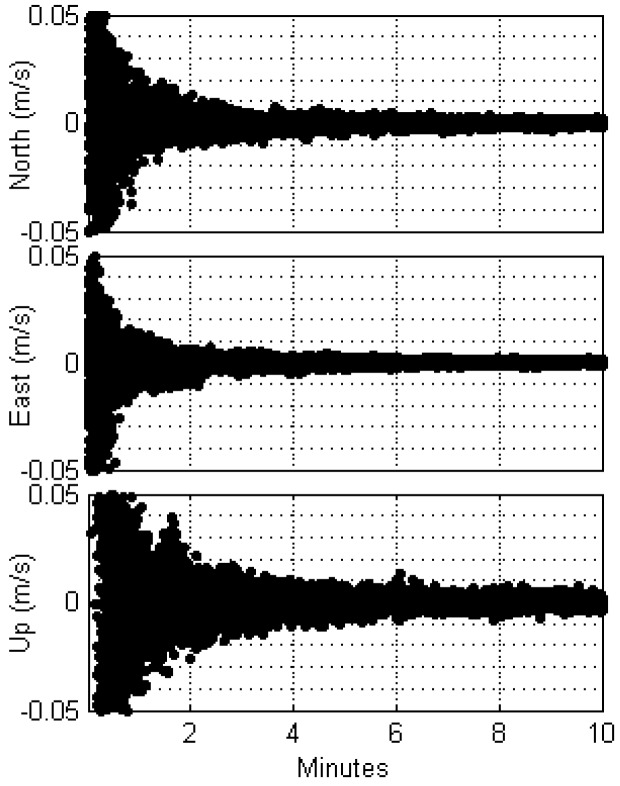
Coordinate rate of change (CROC) for all datasets used in this study. CROCs from 44 hourly datasets were plotted for the first 10 min.

We computed the CROC values of 44 datasets in all three coordinate directions, the results of which are plotted in Figure 3, where the vertical axes have the range of ±5 cm/s. Although the CROC values oscillate significantly at the beginning, they quickly subside in the horizontal direction. In the vertical direction, however, the convergence of CROC is somewhat slow. 

From these patterns, we can conclude that convergence has been reached when CROC remains below 1 cm/s for 10 consecutive epochs. Out of the 44 datasets, the average convergence times were found to be 48, 35, and 74 s for the north, east, and vertical directions, respectively. In the following analysis, we evaluated positioning accuracies after all three directions have converged.

#### 4.1.3. Test Results of Static Positioning

To determine the accuracy of static positioning, we took the first 10-m segment of each of the 44 datasets of SBSA. The positioning accuracy was evaluated in terms of the RMSE of the position estimates with respect to the precise coordinates derived through GIPSY-OASIS. As noted earlier, only those estimates after convergence were used to compute the RMSE. The average horizontal and vertical errors of the 44 datasets were 0.32 and 0.40 m, respectively. It is interesting to note that the accuracy in the vertical direction is quite similar to that of the horizontal direction. 

**Figure 4:**
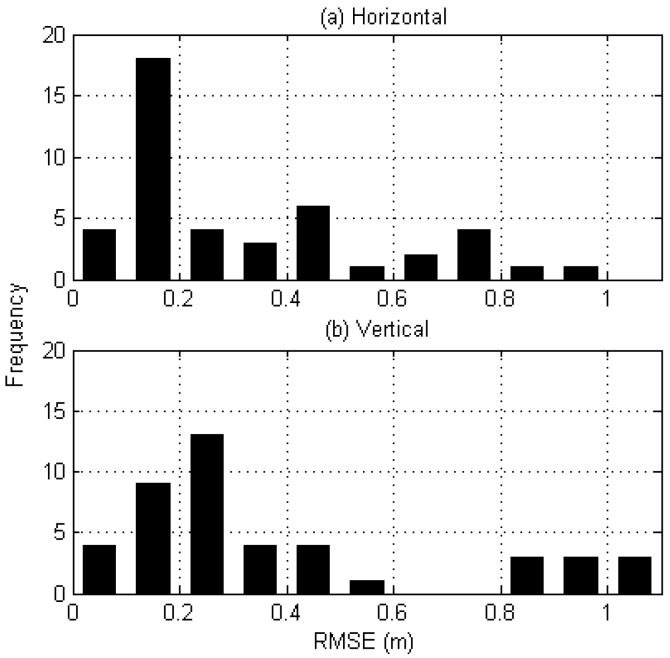
Relative frequency of positioning accuracies after convergence in the (**a**) horizontal and (**b**) vertical directions. A total of 44 test cases are included in the histogram.

Static positioning results are plotted as histograms in Figure 4, where the number of intervals is 11 with a bin size of 0.1 m. The highest frequency for the horizontal direction was observed in a bin of 0.1 to 0.2 m, which corresponds to 41% (18 out of 44). For the vertical direction, bins within a range of 0.1 to 0.3 m account for 50% (22 out of 44). Although all values converge to less than 1 m in the horizontal direction, three cases had an error of larger than 1 m in the vertical direction.

The PPP positioning results were compared to those from Standard Point Positioning (SPP) and DGPS modes. For this comparison, we put up a temporary site at a high school in the city of Incheon, South Korea. The data were collected for three hours with 1-s sampling rate using a ComNav K500 receiver and a u-blox ANN-MS-0-005-0 antenna on 1 November (DOY 305) 2015. In SPP modes, the ionospheric delay was corrected using the Klobuchar model [16]. Of the two components of tropospheric delay, the hydrostatic delay was modeled using the GPT model, and the remaining wet delay was stochastically estimated along the zenith direction [18]. In DGPS modes, the raw data from the receiver were processed by feeding the pseudorange corrections (PRC) from the DGPS reference site [19], EOCH, which is only 9 km away from the high school. Because we do not apply SPP and DGPS with Kalman filtering, we modified our algorithm to conduct least-squares estimations with SSR messages. The results are shown in Table 1. The Positioning accuracy was evaluated with respect to the Virtual Reference System (VRS) Positions. A few South Korean government agencies provide real-time VRS services, and the service offered by the National Geographic Information Institute was used in this study.

As shown in Table 1, the least-squares PPP positioning accuracy was 0.95 and 2.17 m for the horizontal and vertical directions, respectively, which is better than the least-squares SPP by about 0.5 and 1.8 m and DGPS by about 0.5 and 1.3 m. Thus, we can conclude that PPP modes applied SSR correction are more effective than SPP and DGPS modes. The reason for this can be attributed to the fact that the SSR of each error source can represent the individual error characteristics better than the lump-sum magnitude, such as PRC. For the case of the Kalman-filtered PPP, the accuracy improves in the horizontal and vertical directions respectively by 1.2 and 3.5 m than SPP, 1.2 and 3.0 m than DGPS.

### 4.2. Kinematic Positioning

#### 4.2.1. Test Data and GPS Equipment

To evaluate the performance of the SSR messages for the moving platform, we test drove an automobile on about a 4-km stretch of freeway. Test drives were conducted on two separate days: 6 July (DOY 187) and 28 July (DOY 209) 2015. We used a Javad GrAnt-G3 antenna and a Javad Alpha-G3T receiver. It should be noted that the receiver is a single-frequency receiver. C/A observables were taken every second, and the total drive time was approximately 9 min. To compute the positioning accuracies, we applied a splitter to the antenna and fed the same GPS signal to a Septentrio PolaRX3e receiver, through which VRS positions were obtained. As is well known from previous studies, real-time kinematic VRS provides a positioning accuracy of approximately 5 cm in the horizontal direction with respect to RTK [20].

**Figure 5:**
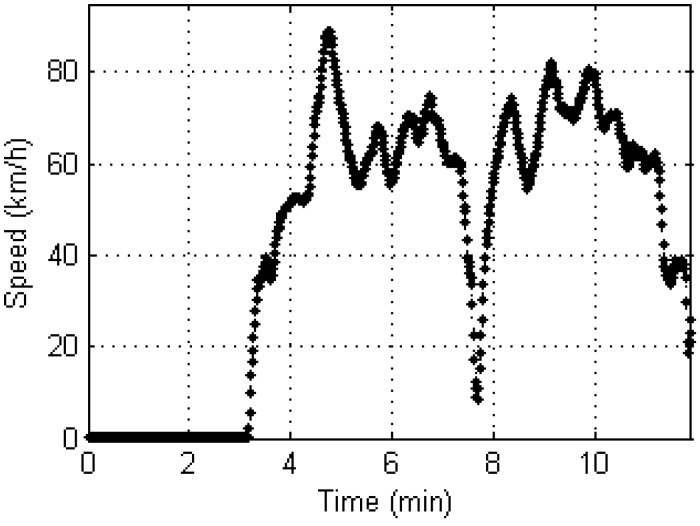
Doppler-based vehicle speed determination for a kinematic test drive.

On both days, the convergence criterion of 1 cm/s was achieved well within 90 s in all directions. The positioning accuracy for the horizontal and vertical directions after initialization was around 0.3 and 0.6 m, respectively. After static initialization, we drove the car at various speeds to see if the positioning algorithm is sensitive to the velocity. The maximum speed was ~90 km/h. We adaptively adjusted the parameters of the system noise Q matrix of the EKF according to the vehicle speed. Instead of using two consecutive positions to derive the velocities, instantaneous Doppler measurements were used. Figure 5 shows the Doppler-based velocity estimates during the entire test period. It can be seen that the speed dropped below 10 km/h at around 7.6 min, which is when the car made a U-turn.

#### 4.2.2. Test Results of Kinematic Positioning

An evaluation of the PPP performance was conducted using two estimation scheme modes. The first is a Kalman-filtered (KF) mode, and the other is an LS mode. The latter mode has an advantage in that no initialization is required. SPP modes were also attempted for a comparison to the LS mode of PPP.

The results of the kinematic positioning accuracy analysis are listed in Table 2. If we consider the average positioning accuracies of SPP for DOY 187 and 209, the RMSE for the horizontal and vertical directions is 2.00 and 3.05 m, respectively. Meanwhile, the best accuracy was achieved using KF mode: 0.53 m for the horizontal direction, and 0.69 m for the vertical direction. Thus, an improvement of ~75% was achieved for all directions. Another notable aspect of SPP is that the levels of accuracy are not consistent between the two test dates. For example, the horizontal accuracy is 2.96 m on DOY 187, and 1.03 m on DOY 209.

**Figure 6:**
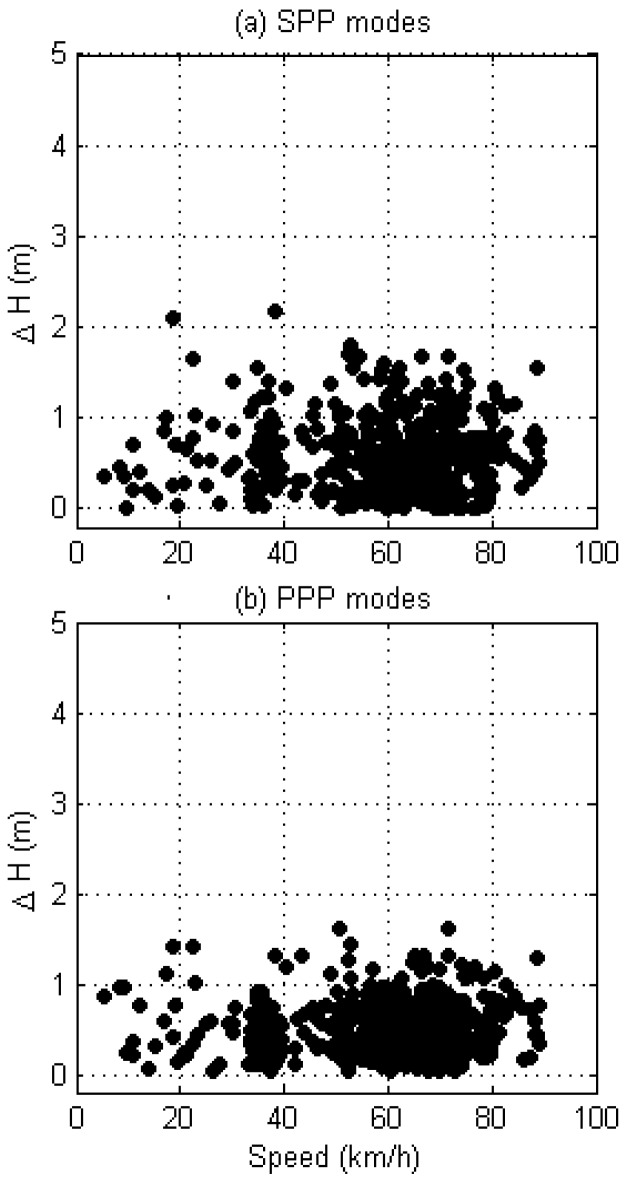
Sensitivity of the positioning error on the vehicle speed in (**a**) SPP and (**b**) PPP modes. The correlation coefficients are (**a**) −0.02 and (**b**) +0.02.

The sensitivity of the positioning accuracy with regard to the vehicle speed was analyzed in Figure 6, which shows the SPP and PPP cases. According to the figure, deviations predominantly reside within the range of 0 to 1 m. As was expected from the results in Table 2, larger scatters were observed in the SPP mode of the kinematic positioning. 

However, no dependency can be found between speed and positioning error. Thus, we can conclude that the developed algorithm is capable of producing a consistent positioning accuracy without regard to the vehicle speed.

## 5. Conclusions

We developed PPP programs to process GPS code-pseudorange observables with real-time SSR corrections, and validated their performance through static and kinematic positioning tests. The performance of PPP algorithm was assessed based on the convergence time and positioning accuracy. With CROC, convergence was considered to be achieved when the rate of change in each direction was less than 1 cm/s. Among the 44 datasets, the average convergence times were found to be 48, 35, and 74 s for the north, east, and vertical directions, respectively. In performance analysis, we evaluated the positioning accuracy after all three directions converged. The proposed algorithm in static modes achieves horizontal and vertical RMSE of 0.32 and 0.40 m, respectively. To examine PPP algorithm for a moving platform, we test drove an automobile on a freeway. The horizontal and vertical RMSE along the test route were 0.53 and 0.69 m, respectively. From the above result, it was verified that the developed PPP algorithm utilizing SSR corrections can be provide reliable sub-meter level accuracy in both static and kinematic modes.

## Figures and Tables

**Table 1:** Comparison of positioning accuracy using SPP-LS, DGPS-LS, PPP-LS, and PPP-KF. LS and KF indicate the least-squares and Kalman filter, respectively.

Coordinate Direction	Positioning Accuracy (m)			
	SPP-LS	DGPS-LS	PPP-LS	PPP-KF
Horizontal	1.40	1.41	0.95	0.22
Vertical	3.94	3.47	2.17	0.47
3-Dimensional	4.18	3.75	2.37	0.52

**Table 2:** Horizontal, vertical, and three-dimensional kinematic positioning accuracies in meters: SPP, LS, and KF. Two test drives were conducted on DOY 187 and DOY 209 in 2015, and ‘average’ indicates two-day averages.

Coordinate Direction	SPP-LS (m)			PPP-LS (m)			PPP-KF (m)		
	187	209	Average	187	209	Average	187	209	Average
Horizontal	2.96	1.03	2.00	0.62	0.76	0.69	0.47	0.59	0.53
Vertical	3.72	2.37	3.05	0.98	0.90	0.94	0.72	0.66	0.69
3-Dimensional	4.75	2.58	3.05	1.16	1.18	1.17	0.86	0.89	0.86

## Appendix A. Sample SSR Message

**Table A1:** Sample satellite orbit corrections provided by SSR messages.

GPS Week Second	PRN	IOD	Radial (m)	Along-Track (m)	Cross-Track (m)
518400	31	12	+0.9803	−0.4988	−0.3716
518400	26	03	+1.2606	+0.0808	−0.2132
518400	16	01	−0.0342	−1.6340	−0.5524
518400	23	72	+0.8714	−1.8136	−0.3956
518400	27	75	+1.1841	+0.7880	−0.6984
518400	21	75	−0.4336	+2.7108	−0.5252
518400	09	98	+1.3928	−2.5820	−0.8112
518400	08	57	+1.5412	−1.1920	+0.0848
518400	19	20	+0.8094	+0.0888	−0.4388
518400	07	86	+1.0294	−1.0124	+0.7224
518400	04	62	+1.2974	+1.6896	−0.9360
518400	11	45	−0.2120	−0.3376	−0.2956
518430	31	13	+1.0437	−0.9324	−0.6548
518430	26	07	+1.1957	+0.4320	−0.1476
518430	16	25	−0.2051	−0.5080	−0.3560
518430	23	73	+0.9296	−2.2384	−0.5168

**Table A2:** Sample satellite orbit corrections provided by SSR messages.

GPS Week Second	PRN	A0 (m)	A1 (m/s)	A2 (m/s²)
518400	31	+3.7972	−0.000067	+0.000000
518400	26	−3.5755	+0.000004	+0.000000
518400	16	+3.0829	+0.000050	+0.000000
518400	23	+3.8690	+0.000089	+0.000000
518400	27	−2.0776	+0.000075	+0.000000
518400	21	+1.8304	−0.000118	+0.000000
518400	09	−0.1725	+0.000097	+0.000000
518400	08	−2.6336	+0.000244	+0.000000
518400	19	+2.1345	+0.000170	+0.000000
518400	07	+1.1194	−0.000030	+0.000000
518400	04	−1.3456	−0.000222	+0.000000
518400	11	−0.4577	−0.000176	+0.000000
518405	31	+3.7974	−0.000061	+0.000000
518405	26	−3.5758	+0.000000	+0.000000
518405	16	+3.0806	+0.000047	+0.000000
518405	23	+3.8690	+0.000092	+0.000000

**Table A3:** Sample satellite bias corrections provided by SSR messages.

GPS Week Second	PRN	C1 (m)	P2 (m)	L1 (m)	L2 (m)
518400	31	+0.00	−1.48	+3.7334	+4.8124
518400	26	+0.00	+2.79	−0.0776	+0.0367
518400	16	+0.00	−1.48	+4.6073	+6.0466
518400	23	+0.00	−2.35	+4.7111	+6.2725
518400	27	+0.00	+1.22	+9.5223	+12.5446
518400	21	+0.00	−1.63	+8.4215	+11.4315
518400	09	+0.00	+0.15	+6.1206	+6.5287
518400	08	+0.00	+2.72	−0.2833	−0.5689
518400	19	+0.00	+2.44	+4.5490	+6.1192
518400	07	+0.00	−0.79	+4.5973	+5.9366
518400	04	+0.00	+2.27	+1.1737	+1.5675
518400	11	+0.00	+0.65	−2.6120	−1.8847
518430	31	+0.00	−1.48	+3.7284	+4.8059
518430	26	+0.00	+2.79	−0.0802	+0.0334
518430	16	+0.00	−1.48	+4.6126	+6.0538
518430	23	+0.00	−2.34	+4.6982	+6.2556

**Table A4:** Sample ionospheric corrections provided by SSR messages.

GPS Week Second	Latitude (Degree)	Longitude (Degree)	Height (m)	STEC (TECU)			
				PRN	STEC	PRN	STEC
518400	+34.000	+126.000	+0.00	04	−20.5668	07	−7.6330
518400	+35.000	+126.000	+0.00	04	−20.3269	07	−7.1539
518400	+36.000	+126.000	+0.00	04	−20.0153	07	−6.5172
518400	+37.000	+126.000	+0.00	04	−19.9662	07	−5.7593
518400	+38.000	+126.000	+0.00	04	−20.3653	07	−4.8207
518400	+34.000	+127.000	+0.00	04	−21.9409	07	−8.6293
518400	+35.000	+127.000	+0.00	04	−21.6632	07	−8.1051
518400	+36.000	+127.000	+0.00	04	−21.3017	07	−7.4215
518400	+37.000	+127.000	+0.00	04	−21.2541	07	−6.6158
518400	+38.000	+127.000	+0.00	04	−21.3993	07	−5.6302
518400	+34.000	+128.000	+0.00	04	−23.0876	07	−9.4855
518400	+35.000	+128.000	+0.00	04	−22.8131	07	−8.8920
518400	+36.000	+128.000	+0.00	04	−22.4792	07	−8.2180
518400	+37.000	+128.000	+0.00	04	−22.2530	07	−7.3896
518400	+38.000	+128.000	+0.00	04	−22.2369	07	−6.3655
518430	+34.000	+126.000	+0.00	04	−20.6940	07	−7.8826
518430	+35.000	+126.000	+0.00	04	−20.4501	07	−7.2972
518430	+36.000	+126.000	+0.00	04	−20.1294	07	−6.5756
518430	+37.000	+126.000	+0.00	04	−20.1123	07	−5.6805

**Table A5:** Sample tropospheric corrections provided by SSR messages.

GPS Week Second	Latitude (Degree)	Longitude (Degree)	Height (m)	ZTD (m)	ZWD (m)
518400	+34.000	+126.000	+0.00	+2.4782	+0.2773
518400	+35.000	+126.000	+0.00	+2.4764	+0.2300
518400	+36.000	+126.000	+0.00	+2.4720	+0.2562
518400	+37.000	+126.000	+0.00	+2.4503	+0.1965
518400	+38.000	+126.000	+0.00	+2.4737	+0.1911
518400	+34.000	+127.000	+0.00	+2.5084	+0.3954
518400	+35.000	+127.000	+0.00	+2.4979	+0.3175
518400	+36.000	+127.000	+0.00	+2.4950	+0.3423
518400	+37.000	+127.000	+0.00	+2.4668	+0.2498
518400	+38.000	+127.000	+0.00	+2.4690	+0.1949
518400	+34.000	+128.000	+0.00	+2.5396	+0.5468
518400	+35.000	+128.000	+0.00	+2.5111	+0.4518
518400	+36.000	+128.000	+0.00	+2.5025	+0.3801
518400	+37.000	+128.000	+0.00	+2.4868	+0.3154
518400	+38.000	+128.000	+0.00	+2.4759	+0.2524
518430	+34.000	+126.000	+0.00	+2.4755	+0.2742
518430	+35.000	+126.000	+0.00	+2.4735	+0.2276
518430	+36.000	+126.000	+0.00	+2.4690	+0.2527
518430	+37.000	+126.000	+0.00	+2.4470	+0.1954
